# Prevalence of Elevated Glycated Hemoglobin Concentrations in the Polycystic Ovary Syndrome: Anthropometrical and Metabolic Relationship in Amazonian Women

**DOI:** 10.14740/jocmr1829w

**Published:** 2014-05-22

**Authors:** Sebastiao Freitas de Medeiros, Marcia Marly Winck Yamamoto, Herica Bernardes Bueno, Danilla Belizario, Jacklyne Silva Barbosa

**Affiliations:** a Department of Gynecology and Obstetrics, Medical School, Federal University of Mato Grosso, 78055-728 Cuiaba, MT, Brazil; b Tropical Institute of Reproductive Medicine and Menopause, 78043-306 Cuiaba, MT, Brazil

**Keywords:** Glycated hemoglobin, Impaired glucose tolerance, Polycystic ovary syndrome

## Abstract

**Background:**

To determine the prevalence of elevated glycated hemoglobin (HbA1c) and to examine its relationship with other carbohydrate metabolic parameter among Brazilian women with polycystic ovary syndrome (PCOS).

**Methods:**

A cross-sectional study including 288 PCOS patients was conducted. Anthropometrical, clinical, biochemical and endocrine parameters were evaluated.

**Results:**

The mean age was 26.92 ± 5.51 years. HbA1c mean concentration was 5.83±1.34%. In 38.54% of patients, HbA1c was ≥ 5.7%. HbA1c was positively correlated with body weight (r = 0.142, P = 0.017), body mass index (P = 0.000), waist:hip ratio (P = 0.000), fat mass (P = 0.000), conicity index (P = 0.000), triglyceride (P = 0.001), C-peptide (P = 0.000), total testosterone (P = 0.003), free testosterone (P = 0.000), free androgen index (P = 0.006) and fasting insulin (P = 0.025). Using the oral glucose tolerance test, HbA1c showed positive correlation with glucose concentrations at any point in time (P < 0.05).

**Conclusions:**

HbA1c was elevated in nearly 40% of PCOS patients and it showed positive correlation with several anthropometric and metabolic factors and androgen levels. The current study provides further evidence that HbA1C is higher in PCOS patients and may have a potential role in the prediction of dysglycemic disease in these women.

## Introduction

Polycystic ovary syndrome (PCOS) is the most common endocrinopathy in women of reproductive age, affecting up to 21% of patients [1]; its prevalence in Brazilian women was not determined yet. Compared with healthy women, patients with PCOS are at a higher risk for coronary heart disease (OR = 1.2 - 12.9), cerebrovascular disease (OR = 2.8 - 3.4), hypertension (OR = 1.4), dyslipidemia (OR = 2.9 - 3.2), myocardial infarction (OR = 2.6 - 4.2), impaired glucose tolerance (IGT) (OR = 2.5), metabolic syndrome (OR = 2.1) and central obesity (OR = 1.9 - 2.4) [2-5]. The risk for type 2 diabetes mellitus (T2DM) is also two- to four-fold higher in patients with PCOS (OR = 2.2 - 3.6). The current screening recommendations for T2DM in patients with PCOS include the measurement of fasting plasma glucose (FPG) and the use of an oral glucose tolerance test (oGTT, 75 g oral dextrose) in cases of obesity, advanced age, personal history of gestacional diabetes or family history of T2DM [6]. The measurement of glycated hemoglobin (HbA1c) is also commonly used to identify non-PCOS individuals at risk of IGT, prediabetes or T2DM [7]. As the use of HbA1c does not require fasting and provides a time-averaged estimate of blood glucose over the preceding 3 - 12 weeks [8], it may be a better indicator of overall glycemia than a glucose concentration at a single point in time [9, 10].

Elevated HbA1c concentrations have been associated with other risk factors for cardiovascular disease (CVD) and the presence of metabolic syndrome in several other non-PCOS clinical conditions and populations [8]. It seems that in patients with or without PCOS, a 1% increase in the absolute HbA1c concentration is associated with a 10-20% increase in CVD risk [8, 11]. The prevalence of elevated HbA1c in women with PCOS has not yet been established worldwide. Previous studies have reported that elevated HbA1c occurs in 10% of PCOS patients in Austria and Turkey [10, 12] and 31% of Korean patients [13]. Given the potential relationships between HbA1c and health-related outcomes in PCOS patients, and the fact that a very few studies have reported on the prevalence and of abnormal HbA1c concentrations in patients with PCOS, the current study aimed to determined the prevalence of elevated HbA1c concentrations in knowledge Brazilian patients with this clinical condition.

## Materials and Methods

The sample consisted of PCOS patients in whom HbA1c levels were measured attending either the Endocrinology or the Reproductive Units at the Julio Muller University Hospital and Tropical Institute of Reproductive and Menopause in Cuiaba, Brazil, until July 2013. The sample size was estimated using an imprecision (i) value of 5%, a mean expected effect of 14% (based on scarce previous studies reporting on the proportion of PCOS patients with an elevated HbA1c) and an alpha level of 5% [10, 14, 15]. Written informed consent was obtained from each patient, as approved by the local Committee for Ethics in Research. Patients were excluded for any of the following reasons: use of sex steroids or insulin-sensitizing drugs over the previous 6 months; thyroxin-stimulating hormone (TSH) concentration ≥ 4.2 µUI/mL and prolactin (PRL) concentration > 25 ng/mL (1,086 nmol/L). Non-classic 21-hydroxylase, 11β-hydroxylase and 3β-hydroxysteroid dehydrogenase (3β-HSD) were excluded as published elsewhere [15].

PCOS diagnosis was performed according to the National Institutes of Health and Rotterdam criteria [1, 15]. Clinical hyperandrogenism was defined as a dichotomous variable using the presence or not of hirsutism in the following body areas: upper lip, chin, chest, upper or lower back, upper or lower abdomen, upper arms and thighs [16]. The free androgen index (FAI) was estimated as total testosterone (nmol/L)/sex hormone-binding globulin (SHBG; nmol/L) × 100. The free estrogen index (FEI) was calculated as follows: 100 × estradiol (nmol/L)/272.1 × SHBG nmol/L) [17]. Ovary transvaginal ultrasound examination was performed using a Voluson machine (Voluson^®^E8, GE Health Care, England) and PCO morphology defined as previous recommendation [18].

### Anthropometric measures

Subjects were weighed on an electronic scale, and height was measured using a Harpender stadiometer (Holtain Limited, Crymych, Dyfed, UK). The waist circumference (WC) was measured at the midway point between the lower rib margin and the iliac crest, and the hip was measured at the widest circumference (location of the greater trochanters). Body mass index (BMI) was calculated as body weight (kg/height (m)^2^). Obesity was defined as BMI ≥ 30 (kg/m^2^) [4]. Lean body mass (LBM) was calculated using the James equation: (1.07 × weight (kg)) - 148 × (weight^2^/(100 × height (m))^2^ [19]. Fat mass (FM) was calculated as: body weight - LBM. Abdominal adiposity was estimated using the conicity index (C index): WC (m)/(0.109 × square root of body weight (kg)/height (m)) [20].

### Biochemical analysis

Triglycerides (TG), high-density lipoprotein cholesterol (HDL-C) and total cholesterol (TC) were measured after a 12-h overnight fast using an enzymatic assay (Wiener Laboratories, Rosario, Argentina). Low-density lipoprotein cholesterol (LDL-C) was calculated as TC - (HDL-C + TG/5) [21]. On a different day, blood was collected for the biochemical and endocrine measurements, followed by a 3-h oGTT and samples obtained basally and at 30, 60, 90, 120 and 180 min after dextrose ingest. Blood samples were drawn between days 3 and 5 in patients with oligomenorrhea or, in amenorrheic patients, in a random day, including progesterone measurement to certify that the blood was collected in follicular phase. The plasma glucose concentration was analyzed using the glucose oxidase technique (Beckman Glucose Analyses, Fullerton, CA, USA). HbA1c was measured using a turbidimetric assay (Wiener Laboratories, Rosario, Argentina). The criteria of elevated HbA1c concentration (≥ 5.7%) with a threshold of ≥ 6.5% to diagnose T2DM were used as recommended by the American Diabetes Association [7]. IGT or prediabetes were defined by a single abnormal parameter as follows: FPG between 100 mg/dL (5.5 mmol/L) and 126 mg/dL (6.99 mmol/L); 2-h oGTT glucose value between 140 mg/dL (7.8 nmol/L) and 199 mg/dL (11.0 nmol/L) [7]. Insulin resistance was defined using fasting insulin levels > 12.2 µU/mL (84.7 pmol/L) [22]; and/or homeostasis model assessment of insulin resistance (HOMA-IR) ≥ 2.8 [23]. The homeostatic model for insulin resistance and tissue sensitivity to insulin (HOMA-IR) was calculated using a free online program [23]: (glucose (nmol/L) × insulin (µU/mL))/22.5.

### Hormone assays

Hormones were measured as described elsewhere [18]. In short, serum luteinizing hormone (LH), follicle-stimulating hormone (FSH), TSH, estradiol, PRL, SHBG and total testosterone levels were measured by electrochemiluminescence assays (Elecsys 2010, Roche Diagnostics GMBH, Mannhein, German). Free testosterone and insulin were measured using a chemiluminescence assay (Siemens Medical Solution Diagnostics, CA, USA) with sensitivity and intra- and inter-assay coefficients of variation as the following: 0.002 pmol/L, 7.0-8.4% for free testosterone and 2 µUI/mL, 4.9-6.4% for insulin.

### Statistical analyses

Data were examined for Gaussian distribution using the Kolmogorov-Smirnov-Lilliefors goodness of fit test, and, where necessary, data were log transformed prior to analysis and subsequently retransformed into the original units for reporting. Anthropometrical, biochemical and endocrine data, presented as mean and standard deviation (SD), were analyzed using the Welch test because equality of variance was not tested. The Z test was used to compare HbA1c status in obese and non-obese PCOS patients. The prevalence rate with 95% confidence intervals (95% CI) was used to compare HbA1c status and other markers of glucose metabolism. The relationships between the HbA1c concentration and anthropometrical, endocrine and metabolic variables were examined using Pearson’s correlation coefficient. Stratified analyses for confounding variables were performed using the Mantel-Haenzel χ^2^ test. All analyses were performed using SPSS for Windows, version 18 (SPSS Inc., Chicago, IL, USA). Statistical significance was set at P ≤ 0.05.

## Results

Of the 288 PCOS patients enrolled, 197 (68.4%) were Caucasian, 41 (14.2%) were African descendants, 41 (14.2%) were of “other” races and nine (3.1%) did not declare their ethnicity. The mean age was 26.92 ± 5.51 years. Sociodemographic and clinical characteristics are shown in Table 1. The BMI was 29.94 ± 7.0 kg/m^2^, FM 30.3 ± 12.54 kg, waist circumference 88 ± 16.31 cm, waist:hip ratio 0.81 ± 0.08 and conicity index 1.16 ± 0.11. The stratified anthropometric features are depicted in Table 2. The overall mean HbA1c concentration was 5.83±1.34%. HbA1c values ≥ 5.7% were present in 111/288 (38.54%), in which 102 (35.4%) had HbA1c between 5.7% and 6.4% and nine (3.12%) had HbA1c ≥ 6.5%. In 177/288 (61.46%), the HbA1c were < 5.7%. After stratification, the associations between HbA1c and other glucose metabolic parameters are shown in Table 3. Overall FPG levels presented mean of 5.11 ± 0.78 mmol/L; its levels were < 5.55 mmol/L in 234/282 (82.9%) patients, between 5.55 mmol/L and 6.99 mmol/L in 39/282 (13.8%) and > 6.99 mmol/L in 9/282 (3.19%) patients. Fasting insulin presented mean of 87.70 ± 2.69 and the levels were > 85 nmol/L in 142/265 (53.5%) patients. The mean HOMA-IR was 1.93 ± 1.21 and the levels were > 2.8% in 57/259 (22.0%) patients, the mean HOMA-β was 128.73 ± 1.52 and < 155% in 166/259 (64.1%) and the C-peptide concentrations were 0.82 ± 0.39 nmol/L and > 1.17 nmol/L in 43/215 (20%) patients.

**Table 1:** Sociodemographic and Clinical Characteristics of Brazilian Polycystic Ovary Syndrome Patients

Parameters	N (288)	%
Age (years)		
14 - 19	28	9.72
20 - 24	64	22.22
25 -29	100	34.72
30 - 34	76	26.38
35 - 39	17	5.90
≥ 40	3	1.04
Social habits		
Etilism	81	28.12
Smoking	18	6.25
None	189	65.62
Physical activity		
Walking	41	14.23
Biking	1	0.34
Other	4	1.39
None	230	79.87
Not recorded	12	4.17
Clinical features		
Infertility	148	51.38
Amenorrhea	57	19.79
Oligomenorrhea	166	57.63
Polymenorrhea	16	5.55
Acne	126	43.75
Hirsutism	149	51.73
Acanthosis nigricans	67	23.26
Striaes	17	5.90
Ultrasound features		
Normal ovary	56	19.44
Polycystic ovary	232	80.56

*One or more clinical feature per patient.

**Table 2:** Distribution of Normal and Abnormal Anthropometrical Characteristics of Polycystic Ovary Syndrome Patients

Variable	n/N	%	Test Z	P*
BMI (kg/m^2^)				
< 30	147/264	56.68		
≥ 30	117/264	44.32	2.611	0.009
Fat mass (%)				
< 32	146/265	55.10		
> 32	119/265	44.90	2.345	0.018
Waist (cm)				
< 88	129/254	50.79		
≥ 88	125/254	49.21	0.354	0.726
W:H ratio				
< 0.80	191/252	75.80		
≥ 0.80	61/252	24.20	11.581	0.000
Conicity index				
< 1.25	112/150	74.67		
≥ 1.25	38/150	25.33	8.544	0.000

*P, two-tailed Z proportion test.

**Table 3:** Analysis of the Association of Glycated Hemoglobin Concentrations With Other Carbohydrate Metabolism Parameters in Polycystic Ovary Syndrome

Variable	HbA1c		Total (n)	PR* (95% CI)	P**
	≥ 5.7 (n)	< 5.7 (n)			
Fasting glucose (nmol/L)					
≥ 5.55	35	13	48		
< 5.55	76	158	234	2.24 (1.68 - 2.76)	0
Total	111	171	282		
Fasting insulin (nmol/L)					
≥ 85	69	73	142		
< 85	37	86	123	1.61 (1.16 - 2.27)	0.003
Total	106	159	265		
Pep-C (nmol/L)					
≥ 1.17	26	17	43		
< 1.17	60	112	172	1.73 (1.19 - 2.32)	0.003
Total	86	129	215		
HOMA-IR					
≥ 2.8	31	26	57		
< 2.8	75	127	202	1.46 (1.04 - 1.95)	0.022
Total	106	153	259		
HOMA % β					
≥ 155	41	52	93		
< 155	65	101	166	1.12 (0.81 - 1.53)	0.510
Total	106	153	259		

*PR: prevalence ratio. **P, two-tailed Z proportion test.

**Figure 1:**
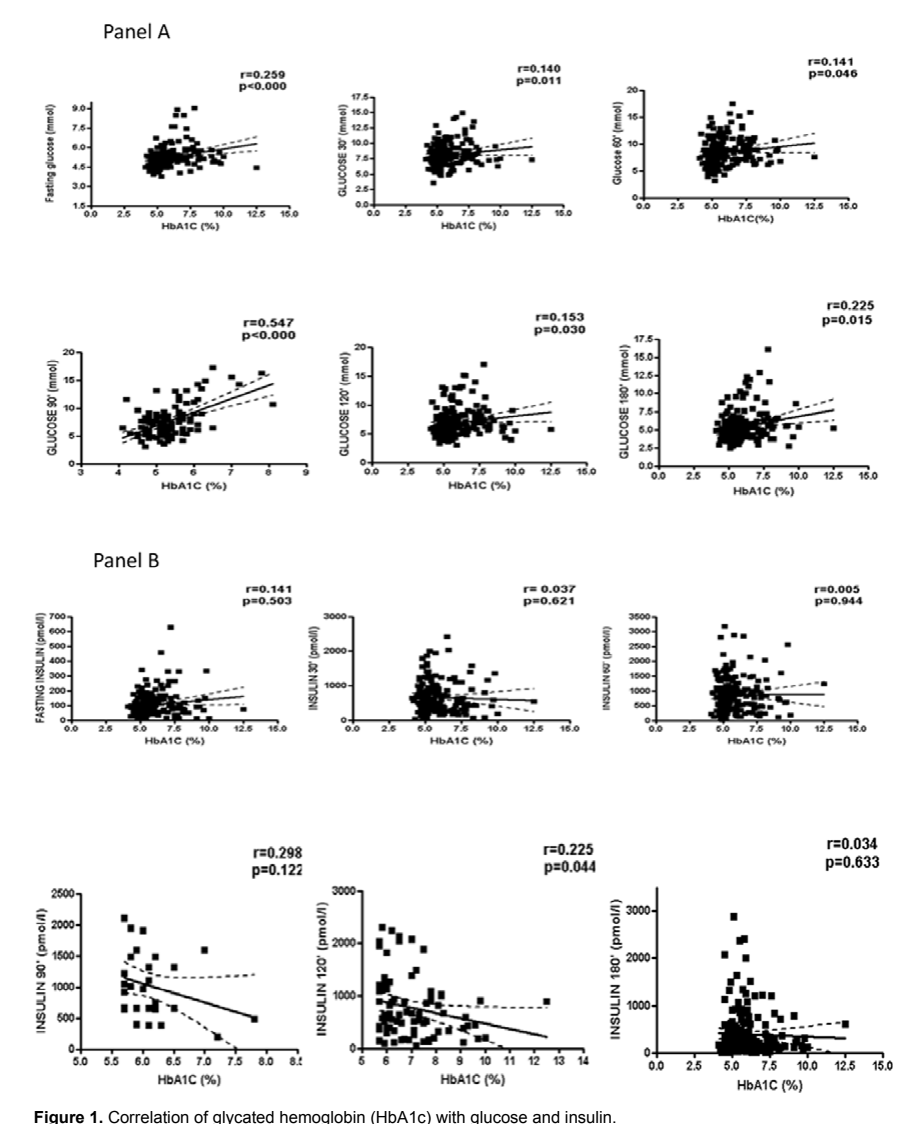
Correlation of glycated hemoglobin (HbA1c) with glucose and insulin.

HbA1c concentrations were positively associated with several anthropometrical (body weight, r = 0.142, P = 0.017; BMI, r = 0.265, P = 0.000; waist:hip, r = 0.271, P = 0.000; FM, r = 0.215, P = 0.000); endocrine (C-peptide, r = 0.238, P = 0.000; total T, r = 0.179, P = 0.003; free testosterone, r = 0.447, P = 0.000; FAI, r = 0.1711, P = 0.018; FEI, r = 0.167, P = 0.055); and metabolic (TG, r = 0.207, P = 0.001) variables. HbA1c was correlated with glucose in the fasting state and any time point after the glucose load (Fig. 1, panel A) but was not correlated with insulin at any of the oGTT time points (Fig. 1, panel B). The distribution of HbA1c levels according the age and BMI is presented in Table 4.

**Table 4:** Stratified Analysis of Glycated Hemoglobin Status According to Age and Body Mass Index in Polycystic Ovary Syndrome

Age (years)	BMI ≥ 30 (n)	BMI < 30 (n)	Total (n)	χ^2^_mh_	P
HbA1c < 5.7%					
< 30	18	31	49		
≥ 30	35	78	113	0.512	0.474
Total	53	109	162		
HbA1c ≥ 5.7%					
< 30	31	10	41		
≥ 30	33	28	61	4.806	0.028
Total	64	38	102		
Total					
< 30	49	41	90		
≥ 30	68	106	174	5.653	0.017
Total	117	147	264		

χ^2^_mh_: Mantel-Haenszel qui-square.

## Discussion

Elevated HbA1c concentrations were found in 38% of PCOS patients, and, after stratification, its levels were not significantly influenced by age or BMI. HbA1c was correlated with a number of variables that are associated with the metabolic syndrome, including fasting glucose and glucose response after oGTT at any point in time. The proportion of PCOS patients with elevated HbA1c levels in the current study was higher than those found in previous reports in other populations. In Korean PCOS patients, also using HbA1c ≥ 5.7% to discriminate normal and high levels, 31% of the patients had elevated HbA1c, which was significantly higher than the results observed in healthy controls (6.6%) [11]. Meanwhile, only 7.6% of Turkish PCOS patients had HbA1c > 5.6% [12], and 8.6% of an older group of Danish PCOS patients presented HbA1c ≥ 6% [24]. Interestingly, 20% of the non-obese patients with PCOS had elevated HbA1c levels in the Korean study, compared to only 6% of the obese PCOS patients [11]. In contrast, the current study found that the levels were twofold higher in obese than non-obese PCOS patients. This difference was no longer present when the sample was stratified according to the HbA1c levels. At this time, it is difficult to determine if the differences between the Korean, Danish, Turkish and Brazilian patients are due to ethnicity or other sample characteristics.

Elevated HbA1c levels have been associated with a more adverse metabolic profile, in PCOS patients in the current study and in the study conducted in Denmark [24]. Although no relationships between HbA1c and insulin either in fasting or after oGTT in the current study was found, such relationships have been reported in the Danish population of PCOS patients [24]. Certainly, more studies are required to confirm the clinical relevance of these data. Significantly higher FAI and free testosterone levels have been reported in women with PCOS and T2DM compared to PCOS women with prediabetes or normal glucose tolerance [10], and significant correlations between HbA1c and FAI and free testosterone have been reported in women with reduced fertility [25]. The study conducted in Danish PCOS patients reported conflicting findings; total testosterone and FAI were not positively correlated with HbA1c levels. The authors suggested that the combination of high HbA1c and low SHBG levels could be better as markers for CVD risk in PCOS patients, based on the presence of inverse relationships between SHBG and HbA1c levels in their sample. Therefore, the influence of age, BMI and endocrinological features on HbA1c concentrations should be examined in future studies and in different populations.

Increased HbA1c levels could potentially be used as a marker of cardiovascular risk in individuals without diabetes [26]. The significant correlations between HbA1c levels and several established anthropometrical predictors of CVD risk in the present study are in agreement with the reported cardiovascular risk in other non-PCOS populations with central obesity, IFG, increased carotid to femoral pulse wave velocity, or low fecund ability rate [5, 27-30]. Within the PCOS population, a recent study reported associations between elevated HbA1c concentrations, larger waist circumferences and higher BMIs in an older group of PCOS patients [24].

One possible limitation of the current study is that social habits that may also affect glycemic status, such as smoking, alcohol use and physical activity, were not completely examined. Second, the study enrolled women who were attending tertiary institutions, and this may have resulted in selection bias and limited the generalizability to the general community. Finally, most of the patients included in the present study met all three Rotterdam criteria for PCOS diagnosis, and this may explain the high prevalence of elevated HbA1c in this sample. In conclusion, HbA1c was elevated in at least one-third of PCOS patients and was positively associated with weight, BMI, waist:hip ratio, FM and androgen levels in the current study. Future clinical studies should be conducted to better understand the potential role of HbA1c as a dysmetabolic variable and a marker of elevated CVD risk in PCOS patients.

## References

[1] March WA, Moore VM, Willson KJ, Phillips DI, Norman RJ, Davies MJ (2010). The prevalence of polycystic ovary syndrome in a community sample assessed under contrasting diagnostic criteria. Hum Reprod.

[2] Wild S, Pierpoint T, McKeigue P, Jacobs H (2000). Cardiovascular disease in women with polycystic ovary syndrome at long-term follow-up: a retrospective cohort study. Clin Endocrinol (Oxf).

[3] Moran LJ, Misso ML, Wild RA, Norman RJ (2010). Impaired glucose tolerance, type 2 diabetes and metabolic syndrome in polycystic ovary syndrome: a systematic review and meta-analysis. Hum Reprod Update.

[4] de Groot PC, Dekkers OM, Romijn JA, Dieben SW, Helmerhorst FM (2011). PCOS, coronary heart disease, stroke and the influence of obesity: a systematic review and meta-analysis. Hum Reprod Update.

[5] Lim SS, Davies MJ, Norman RJ, Moran LJ (2012). Overweight, obesity and central obesity in women with polycystic ovary syndrome: a systematic review and meta-analysis. Hum Reprod Update.

[6] Mani H, Levy MJ, Davies MJ, Morris DH, Gray LJ, Bankart J, Blackledge H (2013). Diabetes and cardiovascular events in women with polycystic ovary syndrome: a 20-year retrospective cohort study. Clin Endocrinol (Oxf).

[7] (2013). Standards of medical care in diabetes--2013. Diabetes Care.

[8] Peters AL, Davidson MB, Schriger DL, Hasselblad V (1996). A clinical approach for the diagnosis of diabetes mellitus: an analysis using glycosylated hemoglobin levels. A clinical approach for the diagnosis of diabetes mellitus: an analysis using glycosylated hemoglobin levels. JAMA.

[9] Nathan DM, Turgeon H, Regan S (2007). Relationship between glycated haemoglobin levels and mean glucose levels over time. Diabetologia.

[10] Lerchbaum E, Schwetz V, Giuliani A, Obermayer-Pietsch B (2013). Assessment of glucose metabolism in polycystic ovary syndrome: HbA1c or fasting glucose compared with the oral glucose tolerance test as a screening method. Hum Reprod.

[11] Kim JJ, Choi YM, Cho YM, Jung HS, Chae SJ, Hwang KR, Hwang SS (2012). Prevalence of elevated glycated hemoglobin in women with polycystic ovary syndrome. Hum Reprod.

[12] Celik C, Abali R, Bastu E, Tasdemir N, Tasdemir UG, Gul A (2013). Assessment of impaired glucose tolerance prevalence with hemoglobin A(1)c and oral glucose tolerance test in 252 Turkish women with polycystic ovary syndrome: a prospective, controlled study. Hum Reprod.

[13] Kim HK, Bae SJ, Choe J (2012). Impact of HbA1c Criterion on the Detection of Subjects with Increased Risk for Diabetes among Health Check-Up Recipients in Korea. Diabetes Metab J.

[14] Lwanga SK, Lemeshow S (1991). World Health Organization: Sample size determination in health studies. A practical manual. World Health Organization, Geneva.

[15] de Medeiros SF, Gil-Junior AB, Barbosa JS, Isaias ED, Yamamoto MM (2013). New insights into steroidogenesis in normo- and hyperandrogenic polycystic ovary syndrome patients. Arq Bras Endocrinol Metabol.

[16] Wild RA, Vesely S, Beebe L, Whitsett T, Owen W (2005). Ferriman Gallwey self-scoring I: performance assessment in women with polycystic ovary syndrome. J Clin Endocrinol Metab.

[17] Sowers M, Derby C, Jannausch ML, Torrens JI, Pasternak R (2003). Insulin resistance, hemostatic factors, and hormone interactions in pre- and perimenopausal women: SWAN. J Clin Endocrinol Metab.

[18] Lujan ME, Jarrett BY, Brooks ED, Reines JK, Peppin AK, Muhn N, Haider E (2013). Updated ultrasound criteria for polycystic ovary syndrome: reliable thresholds for elevated follicle population and ovarian volume. Hum Reprod.

[19] James WPT Department of Health and Social Security and Medical Research Council Group. A repot of the DHSS/MRC group. Her Majesty’s Stationary office: London, 1976.

[20] Valdez R (1991). A simple model-based index of abdominal adiposity. J Clin Epidemiol.

[21] Friedewald WT, Levy RI, Fredrickson DS (1972). Estimation of the concentration of low-density lipoprotein cholesterol in plasma, without use of the preparative ultracentrifuge. Clin Chem.

[22] McAuley KA, Williams SM, Mann JI, Walker RJ, Lewis-Barned NJ, Temple LA, Duncan AW (2001). Diagnosing insulin resistance in the general population. Diabetes Care.

[23] Diabetes Trial Unit: The Oxford Centre for Diabetes, Endocrinology and Metabolism.. Oxford, UK: Oxford University; Available from: http://www.dtu.ox.ac.uk/homacalculator (2013). Accessed 1 May 2013.

[24] Velling Magnussen L, Mumm H, Andersen M, Glintborg D (2011). Hemoglobin A1c as a tool for the diagnosis of type 2 diabetes in 208 premenopausal women with polycystic ovary syndrome. Fertil Steril.

[25] Hjollund NH, Jensen TK, Bonde JP, Henriksen TB, Andersson AM, Skakkebaek NE (1999). Is glycosylated haemoglobin a marker of fertility? A follow-up study of first-pregnancy planners. Hum Reprod.

[26] Adams RJ, Appleton SL, Hill CL, Wilson DH, Taylor AW, Chittleborough CR, Gill TK (2009). Independent association of HbA(1c) and incident cardiovascular disease in people without diabetes. Obesity (Silver Spring).

[27] Lord J, Thomas R, Fox B, Acharya U, Wilkin T (2006). The central issue? Visceral fat mass is a good marker of insulin resistance and metabolic disturbance in women with polycystic ovary syndrome. BJOG.

[28] Gluszak O, Stopinska-Gluszak U, Glinicki P, Kapuscinska R, Snochowska H, Zgliczynski W, Debski R (2012). Phenotype and metabolic disorders in polycystic ovary syndrome. ISRN Endocrinol.

[29] Liang J, Zhou N, Teng F, Zou C, Xue Y, Yang M, Song H (2012). Hemoglobin A1c levels and aortic arterial stiffness: the Cardiometabolic Risk in Chinese (CRC) study. PLoS One.

[30] Kokkoris P, Chantziara C, Toloumis G (2007). Correlation of glycosylated hemoglobin and glucose response to oral glucose tolerance test. Diabetes.

